# Determining free-living daily energy expenditure and physical activity in chronic heart failure: questionnaire—doubly labelled water—motion sensors

**DOI:** 10.1093/ehjopen/oeag048

**Published:** 2026-03-18

**Authors:** Martin Garet, Sylvie Normand, Martine Laville, Jean Michel Gaspoz, Laure Meiller, Valerie Sauvinet, Antoine Da Costa, Jean Claude Barthelemy, Frederic Roche

**Affiliations:** Sainbiose Inserm U1059, Ecole des Mines, University Hospital of Saint Etienne and Jean Monnet University, Campus Santé, F-42023 Saint-Etienne Cedex 2, France; Centre de Recherche en Nutrition Humaine (CRNH) Rhône-Alpes, Centre Européen de Nutrition pour la Santé (CENS), Centre Hospitalier Lyon Sud Pierre Bénite, INSERM U1060, INRA, Université Lyon 1, Hospices Civils de Lyon, 165 Chemin du Grand Revoyet, F-69310 Pierre Benite, France; Centre de Recherche en Nutrition Humaine (CRNH) Rhône-Alpes, Centre Européen de Nutrition pour la Santé (CENS), Centre Hospitalier Lyon Sud Pierre Bénite, INSERM U1060, INRA, Université Lyon 1, Hospices Civils de Lyon, 165 Chemin du Grand Revoyet, F-69310 Pierre Benite, France; Division of Primary Care Medicine, Department of Community Medicine, Primary Care, and Emergency Medicine, University Hospitals, Geneva 14 1211, Switzerland; Centre de Recherche en Nutrition Humaine (CRNH) Rhône-Alpes, Centre Européen de Nutrition pour la Santé (CENS), Centre Hospitalier Lyon Sud Pierre Bénite, INSERM U1060, INRA, Université Lyon 1, Hospices Civils de Lyon, 165 Chemin du Grand Revoyet, F-69310 Pierre Benite, France; Centre de Recherche en Nutrition Humaine (CRNH) Rhône-Alpes, Centre Européen de Nutrition pour la Santé (CENS), Centre Hospitalier Lyon Sud Pierre Bénite, INSERM U1060, INRA, Université Lyon 1, Hospices Civils de Lyon, 165 Chemin du Grand Revoyet, F-69310 Pierre Benite, France; Sainbiose Inserm U1059, Ecole des Mines, Department of Cardiology, University Hospital of Saint Etienne and Jean Monnet University, Campus Santé, F-42023 Saint-Etienne Cedex 2, France; Sainbiose Inserm U1059, Ecole des Mines, University Hospital of Saint Etienne and Jean Monnet University, Campus Santé, F-42023 Saint-Etienne Cedex 2, France; Sainbiose Inserm U1059, Ecole des Mines, University Hospital of Saint Etienne and Jean Monnet University, Campus Santé, F-42023 Saint-Etienne Cedex 2, France

**Keywords:** CHF, Motion sensor, Free-living energy expenditure, Questionnaire

## Abstract

**Aims:**

To evaluate free-living Total and Physical Activity Energy Expenditures (TEE/PAEE) and to assess the validity of the Daily Activity Questionnaire in Heart Failure (DAQIHF) in chronic heart failure (CHF) patients against the doubly labelled water (DLW) and motion sensors methods.

**Methods and results:**

Twenty-nine women/men (12/17) with CHF performed an incremental symptom-limited peak V̇O_2_ test. Free-living TEE and PAEE were estimated with the DAQIHF (TEEquest), motion sensor (Armband® TEEActi) and measured over 2 weeks using DLW (TEEDLW). Resting metabolic rate (RMR) and body composition were assessed with different methods, and peak V̇O_2_ with quality of life were correlated to TEE. Bland–Altman and Student’s *t*-test analyses were used to compare methods. Statistical significance was set for *P* < 0.05. Mean TEE did not significantly differ between TEEDLW and TEEquest (+352.4 kJ.24h^−1^; +5.3%; *P* & NS) for the whole group, nor between women or men, NYHA class, or cardiomyopathy: dilated cardiomyopathy/ischaemic cardiomyopathy. Bland–Altman plots revealed no systematic bias for TEE between methods. In a subgroup of women, TEEquest was significantly higher than TEEacti (*P* < 0.05). RMR estimated from bioelectric impedance overestimated measured RMR (16.4%, *P* & 0.0028). Patients spent 9.4% of their TEE in activities ≥3 metabolic equivalents. Measured peak V̇O_2_ and estimated from the questionnaire were similar (14.1 ± 4.7 vs. 14.8 ± 3.4 mL.min^−1^.kg^−1^; *P* < 0.0001) and were correlated to both TEEDLW and TEEquest (*R* & 0.85 and 0.82, respectively, both *P* < 0.0001).

**Conclusion:**

Free-living TEE and peak V̇O_2_ can be estimated from the DAQIHF in patients with CHF across all activity domains allowing a complete description/assessment of daily physical activity intensities associated with powerful prognostic risk factors.

## Introduction

The promotion of an active lifestyle is still one of today’s main public health concerns in modern countries. It is such an issue that the organization Healthy People 2020 [http://odphp.health.gov/healthypeople/], led by the US government, recognized physical activity (PA) as one of the leading health indicators, which are a measurement of the health of a nation’s population. PA has been shown to play a major role in health status in several populations^1–3^ and is beneficial for the primary prevention of chronic diseases and of all-cause mortality.^4–6^ It is now well established that regular PA delays the onset or progress of different chronic diseases.^7–9^ Integrating PA in primary as well as in secondary prevention has shown to be useful in the management of individuals with chronic heart failure. Pluridisciplinary programmes dedicated to specific populations (i.e. chronic heart failure [CHF]), which include physical training/rehabilitation, reduce the rate of reappearance of symptoms, of rehospitalization and even of mortality following a myocardial infarction.^2–6,9^ Regular PA is therefore highly recommended. The beneficial effects of an increase, even modest, in daily PA in secondary prevention appear essential in CHF patients, known for low levels of daily PA.^9^

Therefore, there is a need to accurately quantify the type and amount of daily PA in patients with CHF for its association with global health and survival. As the need for comprehensive assessments of daily PA has grown, the limitations of existing methods used in patients with CHF have become evident. Recent reviews pointed out the strengths and pitfalls of various techniques, such as motion sensors, questionnaires and gold standard quantification methods, like the use of doubly labelled water (DLW).^10–12^ Current direct and indirect methods measuring or estimating energy expenditure and PA in free-living conditions vary in terms of accuracy and feasibility. Even though the doubly labelled water technique is the most accurate in field research and has been used in patients with CHF,^13–15^ its high cost makes it maladjusted to large population surveys or routine use. Furthermore, information concerning the pattern of activities or the details of living habits cannot be obtained without activity diaries and these are time-consuming and require the complete cooperation of the person throughout the collection period. Heart-rate monitoring is an alternative method but does not systematically provide accurate estimates of total daily energy expenditure (TEE) and is not adapted to individuals under medication for chronic heart failure and possibly with arrhythmias, chronotropic insufficiency or other cardiac disturbances.

Wearable motion sensors have been widely accepted as useful and practical for wearable devices to measure and assess PA in either clinical/laboratory settings or free-living environments.^16^ By contrast, PA questionnaires remain the instrument of choice for PA surveys in large population studies,^17^ or to assess TEE because of their practical value, easiness of use, and low cost. However, most available questionnaires better reflect variations in high-intensity PA, which limits their use in chronically disabled populations, where light or moderate-intensity PA is mostly performed.^10^ Moreover, PA questionnaires need to be reliable and sensitive to small variations in daily PA or energy expenditure in order to be used at the individual level in clinical practice to ensure an accurate follow-up. This is of particular importance from the patient’s perspective in order to assess the gain obtained by the efforts made in modifying his daily level of PA to break a deconditioning process. Thus, an adequate PA questionnaire should reflect both the type and quantity of usual PA and their respective modifications in a target population and their results be expressed in standardized units of energy expenditure in order to allow a dose–response analysis between PA and health-related issues.

We proposed such a questionnaire providing a complete picture of a patient’s usual activities and validated its use in a population of CHF patients, a population known to present low levels of daily PA and energy expenditures.^18^ This questionnaire showed a high level of reliability, reproducibility and inter-observer validity in this population.^18^ In a further pilot study, the estimation of TEE from the questionnaire was validated against the gold standard method for TEE assessment in free-living conditions, namely the doubly labelled water method, but in a limited number of patients.^19^ More interestingly, the results from the questionnaire presented a high sensitivity to changes in daily PA, as modifications in TEE or physical activity energy expenditure (PAEE) were correlated to concomitant modifications in peak V̇O_2_ (ref.^18^, considered as the best measure of physical fitness^6,20^ and an excellent integrated measure of function in these and other ones, such as the elderly.^17,20–23^

Since a major goal is to improve daily PA levels, often limited by symptoms in chronic heart failure, it now appears interesting to assess the validity and reliability of the questionnaire across all activity domains, and to provide a complete thorough description of daily PA and energy expenditures in this population, including sedentary behaviours, for a precise follow-up of the patient. Of note, the beneficial effects of increasing PA in patients with CHF can also be noticed at a psychological level, with a reduction in associated depressive disorders and an improvement in self-esteem and quality of life.^24–27^

Thus, the aims of this study were, in a population of patients with CHF; (1) to assess the validity of the DAQIHF questionnaire against DLW and motion sensor estimates of TEE and PAEE details; (2) to assess the reliability of the questionnaire by administering it on 2 occasions; (3) to provide a detailed description of patterns of PA; and (4) to evaluate the association between the amount of PAEE and quality of life.

## Methods

### Population

Twenty-nine patients with stable chronic heart failure (mean age: 60.0 ± 12.0 years, mean left ventricular ejection (LVEF) = 33.1 ± 9.3%; women/men: 12/17) were recruited from the University Hospital of Saint-Etienne, France. Mean LVEF was determined by echocardiography. Fourteen patients suffered from DCM and 15 patients from ICM. At the time of inclusion, patients were haemodynamically stable, free of oedemas and were taking two or more of the following medications: diuretics, angiotensin-converting enzyme inhibitors, betablockers or digoxin; no patient was on anti-arrhythmic drugs. Participants were defined by the NYHA functional scale as class I (*n* = 3), class II (*n* = 7), class III (*n* = 16), and class IV (*n* = 3). All subjects were free of cigarette use, none had significant pulmonary disease or thyroid dysfunction, and all were stopped by dyspnoea or leg fatigue on incremental exercise testing. No patient was wasted, cachectic, or with morbid obesity. The study protocol complied with the principles outlined in the declaration of Helsinki. Written informed consent was given by all participants and the study was approved by the regional ethical committee. The study was declared on Clinical Trials (ClinicalTrials.gov Identifier: NCT00676390).

## Material and methods

### Design of the study

Patients came to the hospital for a routine check-up of their cardiac function with exercise testing. On this occasion, they were asked to participate in the study. Seven to 10 days later, they returned to the hospital, filled a physical activity questionnaire (DAQIHF), as well as a quality of life questionnaire (Duke QoL questionnaire). Blood (25 mL), urine and salivary samples (30 mL/10 mL each, immediately frozen at −80°C) were collected after a 12-h overnight fast for the assessment of nutritional status, blood natriuretic peptide (BNP) concentration, and baseline urine concentration for assessment. After the ingestion of the dose, urine/salivary samples were taken at 3 and 4 h (for the measurement of total body water), and in the morning (first void) of the 7th following day and again on the 14th day, for the determination of TEE. The dose was determined based on body weight, in accordance with a standardized protocol described below. During the first hour following the sample ingestion, resting metabolic rate (RMR) was measured (see below) during 60 min and estimated from standard equations. Following RMR measurement, in a subgroup of 12 women, the triaxial motion sensor (SenseWear Armband, BodyMedia, Pittsburgh, PA, USA) was initialized using the participant’s personal information and adjusted to snugly fit on the triceps of the participant’s left arm, following the manufacturer’s recommendations. Patients were asked to wear the sensor 24 h a day (besides bath/shower) during 7 days and returned the sensor, along with salivary and urine samples (1st morning void), on the 7th day following the initial ingestion of DLW. On the 14th day, patients returned to the hospital for final salivary/urine sample collection and filled again both questionnaires (DAQIHF and Duke QoL questionnaire).

As daily PA and energy expenditure are subject to change with the environment and seasons, the distribution of the population sample across NYHA classes within each season was made similar (NYHA I and II classes: 50% were included during the autumn/winter period and 50% during the spring/summer period; NYHA III and IV classes: 55% were included during the autumn/winter period and 45% during the spring/summer period; NYHA III class: 55% were included during the autumn/winter period and 45% during the spring/summer period).

### Exercise test with metabolic gas exchange analysis

Resting, ventilatory threshold and peak workload, MET as well as V̇O_2_, V̇E/V̇O_2_, V̇E/V̇CO_2_, V̇E, V̇CO_2_ MET, *Q*_R_, and heart rate were established from a symptom-limited exercise test performed on an electronically calibrated cycle ergometer under control of a cardiologist and specialized technicians. After a 2 min warm-up at 10 W, power was increased by 10 W every minute at a cycling frequency of 60 rpm. Standard 12-lead electrocardiograms were obtained at rest, each minute during exercise and in the recovery phase. Blood pressure was monitored using a standard sphygmomanometer, at rest and every 2 min during exercise and recovery. Levels of mixed expired oxygen (O_2_), mixed expired carbon dioxide (CO_2_), and expired volume were analysed at rest and every 15 s during the protocol using the Medical Graphic Corporation Metabolic Cart (Saint Paul, Minnesota, USA). Instruments were calibrated before each test. Peak V̇O_2_ was defined as the highest V̇O_2_ occurring during the last stage of maximal exercise. A minimum of 3 min was allowed before starting each test to ensure that stable resting measurements were obtained. Each subject was encouraged to exercise until exhaustion and none of them stopped exercise due to angina or claudication. The criteria for reaching the peak V̇O_2_ were a respiratory exchange ratio above one and/or dyspnoea and/or physical exhaustion.

### Measurement of resting metabolic rate

Subjects were asked to come to the laboratory at 8:00 a.m. after an overnight fast. Participants were supine and resting quietly by avoiding speaking and minimizing their movements and RMR was measured continuously, breath by breath, by indirect calorimetry (RMR_calo_) for 60 min under thermoneutral temperature conditions, using the breathing mask of the Medical Graphic Corporation Metabolic Cart (Saint Paul, Minnesota, USA). RMR was also estimated from Bioelectrical Impedance Analysis (RMR_BIA_; Nutriguard NS, Pöcking, Germany) and the equation of Harris and Benedict (RMR_HB_). RMR was also estimated from the questionnaire (RMR_quest_ corresponding to ‘Rest energy expenditure’).

### Measurement of daily energy expenditure

#### Doubly labelled water method

After a baseline urine sample, DLW was administered orally 10% H_2_^18^O (0.15 g.kg^−1^ body mass) mixed with 99.8% d’^2^H_2_O (0.075 g.kg^−1^ body mass). Samples of urine were taken at 3 and 4 h after ingestion (for the measurement of total body water) and in the morning (first void) of the 7th day and again on the 14th day for the determination of daily energy expenditure (DEE). Analyses were performed by isotopic ratio mass spectrometry. Samples were analysed for isotopic enrichment in deuterium using the offline zinc reduction procedure and for ^18^O using the CO_2_ equilibration technique.

Samples were analysed in triplicate for ^2^H_2_O (SD & ± 2 ç). After zinc reduction, 2 μL of urine sample was introduced into a microconical glass insert placed in a glass reaction vessel filled with 80 mg of zinc reagent. These vessels (under vacuum) were heated in a 500°C oven for 30 min shortly before being analysed. Samples were analysed in duplicate for H_2_^18^O (SD: ± 0.2 ç) by CO_2_ equilibration. Two millilitres of urine sample, in a special 15 mL glass container, were isotopically equilibrated with CO_2_ for 4 h in an Isoprep 18 (Fisons, United Kingdom), then CO_2_ was dried by passing the CO_2_ stream through an ethanol-frozen trap (−80°C) before analysis.

#### Motion sensor analyser

The SenseWear Armband (BodyMedia Inc.) is an activity monitor worn on the upper limbs to measure physical activities. The SenseWear Armband combines a dual-axial accelerometer to measure motion and multiple sensors to measure skin temperature, heat flux, and galvanic skin response. This system can report TEE, METs, total number of steps, and sleep duration. Compared with indirect calorimetry, the SenseWear Armband accurately assessed EE across slow to normal walking, but showed underestimation of EE during increased walking speeds.^28^ A subgroup of 12 women were asked to wear the SenseWear Armband during 7 days between days one and seven, without changing anything to their regular usual activities. Motion sensor data from male participants were excluded from analysis due to technical issues with sensor compliance and data quality that prevented reliable measurements in this subgroup. Data obtained from the SenseWear Armband were retained only if the average wearing time reached more than 22 h a day for at least 5 successive days.

#### Physical activity questionnaire—description of daily physical activity

A detailed description of the DAQIHF is available elsewhere.^18^ Briefly, the DAQIHF is a structured questionnaire specifically adapted to patients with CHF. It systematically explores the patient's activities across a typical week, covering all periods of the day (sleeping, resting, light activities, moderate activities, and intensive activities). For each activity domain, patients report the average time spent per day. The questionnaire, adapted to patients with CHF, provides an estimation of peak V̇O_2_ and an individual complete qualitative and quantitative picture of their mean daily activities, with a calculation of their TEE corrected for age, weight, severity of their condition and autonomy. In this study, physical activity (PAEE) and total energy expenditure were analysed according to the duration and intensity of activity periods as presented below:

TEE: Total Energy Expenditure

PAEE: Physical Activity Energy Expenditure during the awakened period.

Rest: Sleeping, naps, doing nothing.

PA_low_: Physical activity strictly below 3 METs (metabolic equivalent).

PA_high_: Physical activity from 3 to 5 METs.

PA_intensive_: Intensive activities (strictly above 5 METs).

In this study, the results of the questionnaire were computerized once with the correction coefficients for age, weight, severity of the condition and autonomy, and once without them, in order to evaluate the validity of these calculation coefficients.

### Quality of life questionnaire

The quality of life of the patients was assessed with the Duke quality of life questionnaire already used in CHF.^29^

### Body composition and anthropometric characteristics

Anthropometric characteristics include height, weight and body mass index (BMI) in all participants. Body composition assessment was performed using Bioelectrical Impedance Analysis (BIA) and the doubly labelled water dilution technique. For BIA, resistance and reactance were measured twice by BIA generator with four surface electrodes placed on the left wrist and ankle (Nutriguard NS, Pöcking, Germany) and were used to mathematically derive fat-free mass (FFM) and fat mass (FM). For the doubly labelled water dilution technique, 2 samples were analysed with a 14-days time interval.

### Statistics

Results are given as mean ± SD for continuous variables. Normality of the distributions was assessed with a Kolmogorov–Smirnov test.

Differences in TEE obtained between the DAQIHF and the DLW method were assessed with a Bland and Altman test.^30^ Differences from the questionnaire with and without correction coefficients were assessed using a paired Student *t*-test. Differences in RMR obtained from DLW or BIA or calorimetry or equations, as well as differences in body composition assessed from DLW or BIA, as well as differences in peak *V’*O_2_ assessed or estimated from the questionnaire, were assessed using a paired Student *t*-test.

Differences in daily energy expenditure and its intensity dimensions (DAQIHF vs. SenseWear), time spent in activity levels, activity scores and QoL variables between patients within different NYHA classes and between ICM/DCM groups were based on unpaired Student’s *t*-tests. The relationships between TEE or activity intensity levels and QoL were assessed with simple regression analysis with QoL as the dependant variables. Multiple comparisons were performed without adjustment for family-wise error rate; therefore, results should be interpreted with appropriate caution regarding potential Type I error inflation. Statistical significance was set for *P* < 0.05 for all analyses.

## Results

### Population

Characteristics of the population are presented in *Table 1*. All patients successfully completed the study but one, for whom no data were available for RMR_calo_. There were no significant differences in age, anthropometric characteristics, LVEF, BMI, peak V̇O_2_, BNP, C-reactive protein, TEE, PAEE, or any intensity dimension of TEE within women and men, or between the overall population of patients with ICM/DCM (*Table 1,* P = NS all). Data were therefore pooled for these subgroups. Height (*P* = 0.0016), weight (*P* = 0.0192), and peak V̇O_2_ (*P* = 0.0063) were significantly higher in men compared to women. For peak V̇O_2_ assessment, patients stopped because of exhaustion with an average maximal heart rate reaching 107.5 ± 21.0 b.p.m. corresponding to 64.6 ± 12.5% of the predicted maximal heart rate. Average maximal respiratory exchange ratio was 1.15 ± 0.1, suggesting a maximal effort in all. No significant differences were observed between NYHA classes but for age (NYHA II vs. III, *P* = 0.0014; III vs. IV, *P* = 0.0014), peak *V’*O_2_ (NYHA II vs. III, *P* = 0.0023; II vs. IV, *P* = 0.0305; III vs. IV, *P* = 0.0059) and quality of life (NYHA II vs. III, *P* = 0.01).

**Table 1:** Characteristics of the population

		Age (years)	Height (cm)	Weight (kg)	BMI (kg.m^−2^)	NYHA	LVEF (%)	BNP (ng.L^−1^)	C-reactive protein (mg.L^−1^)	Peak *V’O_2_*(mL.min^−1^.kg^−1^)
Women (*n* = 12)	ICM (*n* = 6)	61.4 ± 8.9	159.2 ± 5.1	64.6 ± 13.9	25.5 ± 5.6	II = 2 III = 3 IV = 1	28.3 ± 2.4	820.2 ± 865.5	2.8 ± 2.8	10.7 ± 2.3
	DCM (*n* = 6)	56.6 ± 15.5	165.6 ± 7.6	73.6 ± 11.2	26.9 ± 4.3	II = 2 III = 3 IV = 1	36.3 ± 10.5	295.5 ± 447.5	2.4 ± 0.7	11.6 ± 3.9
Men (*n* = 17)	ICM (*n* = 9)	58.6 ± 13.7	172.4 ± 6.2	78.4 ± 12.2	26.3 ± 3.5	II = 3 III = 5 IV = 1		836.0 ± 0.0	4.6 ± 0.0	15.2 ± 3.7
	DCM (*n* = 8)	61.7 ± 11.6	172.9 ± 7.9	85.3 ± 8.9	28.5 ± 2.4	I = 1 II = 3 III = 4	37.5 ± 10.6	151.3 ± 96.2	4.2 ± 2.4	16.9 ± 4.9
Overall (*n* = 29)	ICM (*n* = 15)	60.3 ± 11.3	167.1 ± 8.4	71.0 ± 14.8	25.3 ± 4.7	II = 5 III = 8 IV = 2	25.6 ± 6.3	872.6 ± 718.9	3.2 ± 2.3	12.8 ± 4.2
	DCM (*n* = 14)	61.7 ± 10.2	169.3 ± 8.3	81.2 ± 11.2	28.3 ± 2.9	I = 1 II = 5 III = 7 IV = 1	38.0 ± 9.4	136.8 ± 160.0	3.7 ± 2.1	15.2 ± 5.0
Total (*n* = 29)	ICM + DCM	61.1 ± 10.5	168.3 ± 8.3*	76.6 ± 13.7*	26.9 ± 4.1	I = 1 II = 10 III = 15 IV = 3	33.2 ± 10.2	480.1 ± 615.4	3.5 ± 2.1	14.1 ± 4.7*

*P < 0.05 between women and men.

DCM = dilated cardiomyopathy; ICM = ischaemic cardiomyopathy.

### TEE measurements, validity of the questionnaire

Results of free-living TEE_DLW_, TEE_acti_, and TEE_quest_ are presented in *Table 2*. TEE_quest_ slightly overestimated TEE_DLW_ (mean difference = + 352.4 kJ.24h^−1^; +5.3%) and was significantly higher than TEE_acti_ in the subgroup of 12 women (mean difference = + 1537.5 kJ.24h^−1^; +14.2%; *P* < 0.05). In this subgroup, TEE_acti_ was slightly higher than TEE_DLW_ (mean difference = + 8.5 kJ.24h^−1^; +0.8%; *P* = NS). Considering TEE_DLW_ as the referent value, individual differences ranged from −21.0% to +40.2% in TEE_quest_ for the whole population and from −21.4% to +32.4% in TEE_acti_ in women.

**Table 2:** Energy expenditure values in women and men from the Armband sensewear, the doubly labelled water method and the DAQIHF, with and without correction values

	TEE_DLW_ (kJ.24h^−1^)	TEE_acti_ (kJ.24h^−1^)	PA_highacti_ (kJ.24h^−1^)	TEE_quest_ (kJ.24h^−1^)	PA_highquest_ (kJ.24h^−1^)
Women (*n* = 12)	8036.2 ± 930.9*	8232.3 ± 1292.1	1275.4 ± 1117.4	9325.9 ± 1333.7*	1075.8 ± 672.7
Men (*n* = 17)	11 633.7 ± 2392.8*			11 148.7 ± 2557.7*	1360.5 ± 1107.4
Total (*n* = 29)	10 220.4 ± 2629.6	8232.3 ± 1292.1^a^	1275.4 ± 1117.4	10 419.6 ± 2309.6 ^a^	1246.6 ± 954.2

*P < 0.05 between women and men.

^a^P = 0.0056 between TEE_quest_ and TEE_acti_ in women.

**Figure 1:**
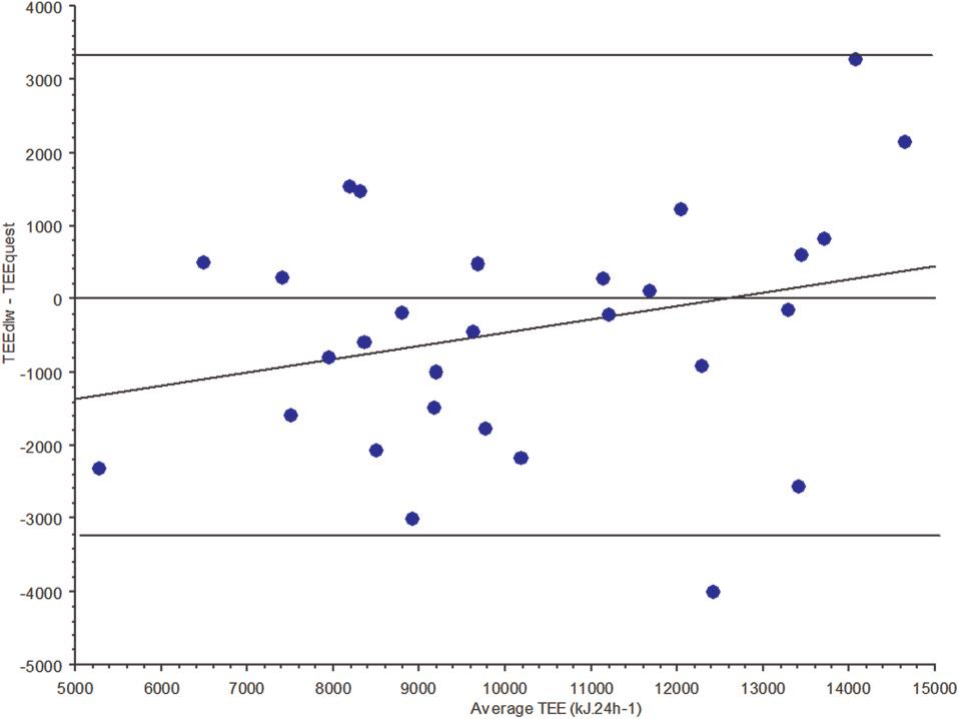
Agreement between TEE estimation (DLW method vs. questionnaire according to Bland and Altman plot).

Comparing TEE_DLW_ and TEE_quest_, Bland-Altman plots revealed no systematic bias for TEE between the two methods (*Figure 1*). There was no significant difference in TEE_DLW_ vs. TEE_quest_ for the whole group (*P* = NS), nor when considering ICM/DCM.

When calculated without the correction coefficients, there were significant differences between TEE_quest_ (12 583.5 ± 3235.7 kJ.24h^−1^) and TEE_DLW_ (10 220.4 ± 2629.6 kJ.24h^−1^, *P* < 0.02) and TEE_quest_ with the correction coefficients (*P* < 0.02).

### Reliability of the questionnaire

**Figure 2:**
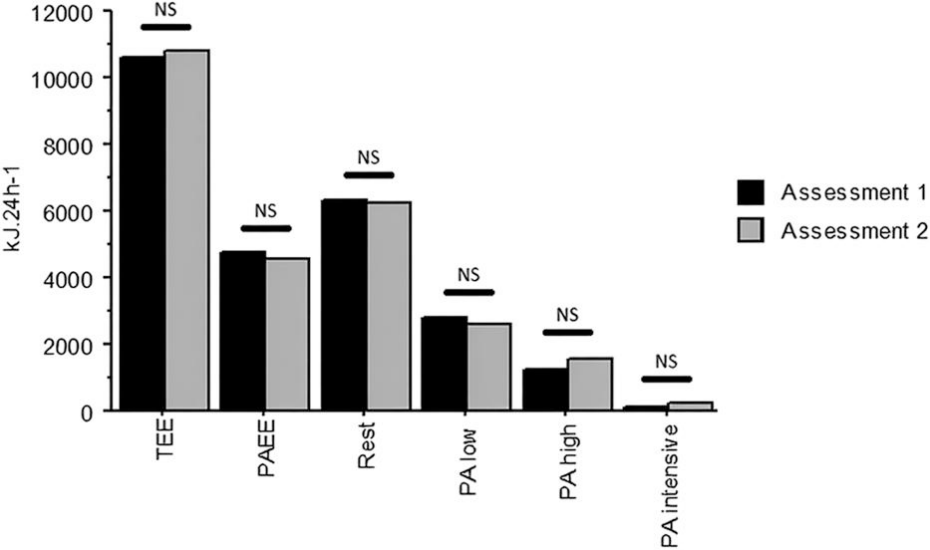
Reproducibility results for TEE and TEE dimensions between two assessments with a 2-week interval period. No significant differences were found in any dimension of TEE. For abbreviations see text.

Paired Student’s *t*-test revealed no significant differences between the two administrations of the questionnaire in TEE or any TEE dimension investigated. *Figure 2* illustrates the concordance in all areas of activity for the 29 patients. *Table 3* shows the prevalence of TEE and its dimensions during the first and second administration of the questionnaire, mean differences between the two administrations, comparison based on a paired *t*-test and correlation coefficients between the first and the second administration.

**Table 3:** Test-retest results of the two administrations of the questionnaire. For abbreviations see text

	Assessment 1		Assessment 2		Quest. 2 − Quest. 1 Difference		*P* ^a^	*R*	*P* ^b^
	Mean	SD	Mean	SD	Mean	SD			
TEE (kJ.24h^−1^)	10 282.2	2222.3	10 559.5	2545.8	128.6	989.3	0.5053	0.923	<0.0001
PAEE (kJ.24h^−1^)	4172.7	1528.3	4327.3	1540.3	18.3	874.2	0.9140	0.834	<0.0001
Rest (kJ.24h^−1^)	6170.6	1804.5	6193.5	1454.5	78.2	805.0	0.6179	0.892	<0.0001
PA_low_ (kJ.24h^−1^)	2882.7	941.2	2621.2	763.4	−319.3	842.7	0.0597	0.531	0.0037
PA_high_ (kJ.24h^−1^)	1222.3	961.6	1459.5	1156.9	171.3	450.0	0.0586	0.926	<0.0001
PA_intensive_ (kJ.24h^−1^)	128.5	195.4	223.4	426.6	85.3	417.5	0.2979	0.279	0.0005

*P* ^a^ = *P* value based on paired Student’s *t*-test.

*P* ^b^ = *P* value based on correlation coefficient.

*R* = Pearson product moment correlation coefficient.

### Physical activity dimensions

*Table 3* presents the values of all TEE dimensions explored with the questionnaire for the whole group. As the Armband allows an exploration of dimensions of TEE, it has to be noticed that in the women subgroup, no significant differences appeared in PAEE and activities above 3 METs (PA_highquest_ vs. PA_highacti_, 1075.8 ± 672.7 kJ.24h^−1^ vs. 1275.4 ± 1117.4 kJ.24h^−1^ respectively; *P* = NS) nor in activities above 5 METs (*P* = NS) but in resting and low-intensity activities below 3 METs (PA_low_, Rest and sleep, *P* < 0.05).

Qualitatively, results from the questionnaire reported that patients spent only 9.4% of their TEE in activities above the 3 METs, threshold known to be associated with prognostic factors, and only 0.9% of their TEE above 5 METs. Regarding the questionnaire’s results, there was a modest significant difference between women and men only in TEE (9325.9 ± 1333.7 kJ.24h^−1^ vs. 10 957.3 ± 2500.1 kJ.24h^−1^, respectively; *P* = 0.0496) but not in any dimension. Women were more active than men (PAEE = 43.7% and 37.5% of TEE in women and men, respectively) but spent more energy in low-intensity activities (Rest + PA_low_ = 96.1% and 83.2% in women and men, respectively; *P* = 0.0151).

TEE slightly decreased with NYHA class and was significantly different between classes II and III (*P* = 0.0278) and II and IV (*P* = 0.0275) but not between classes I and II or III and IV (*P* = NS). PAEE decreased between classes II and III only (*P* = 0.0393). When comparing PA_high_ and PA_intensive_ between values obtained from the questionnaire and from the Armband, no significant differences appeared within each NYHA class (*P* = NS all).

### Peak V̇O_2_ and peak V̇O_2_ estimation from the questionnaire

Results of peak V̇O_2_ are presented in *Table 1* and were independent from ICM/DCM condition and age, and related to LVEF (*R* = 0.68, *P* = 0.009). Average maximal heart rate was 107.5 ± 21 b.p.m. corresponding to 64.6 ± 12.5% of the predicted maximal heart rate. Average maximal respiratory exchange ratio was 1.15 ± 0.1, suggesting a maximal effort in all. Significant differences were noticed in peak V̇O_2_ between NYHA classes II and III (16.4 ± 5.5 mL.min^−1^.kg^−1^ vs. 12.4 ± 2.2 mL.min^−1^.kg^−1^; *P* = 0.0023), II and IV (16.4 ± 5.5 mL.min^−1^.kg^−1^ vs. 8.3 ± 0.5 mL.min^−1^.kg^−1^; *P* = 0.0305) and III and IV (*P* = 0.0059).

Estimated peak V̇O_2_ when calculated from the questionnaire with the correction coefficients (14.8 ± 3.4 mL.min^−1^.kg^−1^) was not significantly different from peak V̇O_2_ obtained from exercise testing (*t*-value = −0.5; *P* = NS). However, when calculated without the correction coefficients (20.7 ± 2.5 mL.min^−1^.kg^−1^), estimated peak V̇O_2_ from the questionnaire was significantly different from peak V̇O_2_ obtained from exercise testing (*t*-value = −4281; *P* < 0.0001) and from peak V̇O_2_ estimated from the questionnaire, including the correction coefficients (*t*-value = −6.154; *P* = 0.0003).

**Figure 3:**
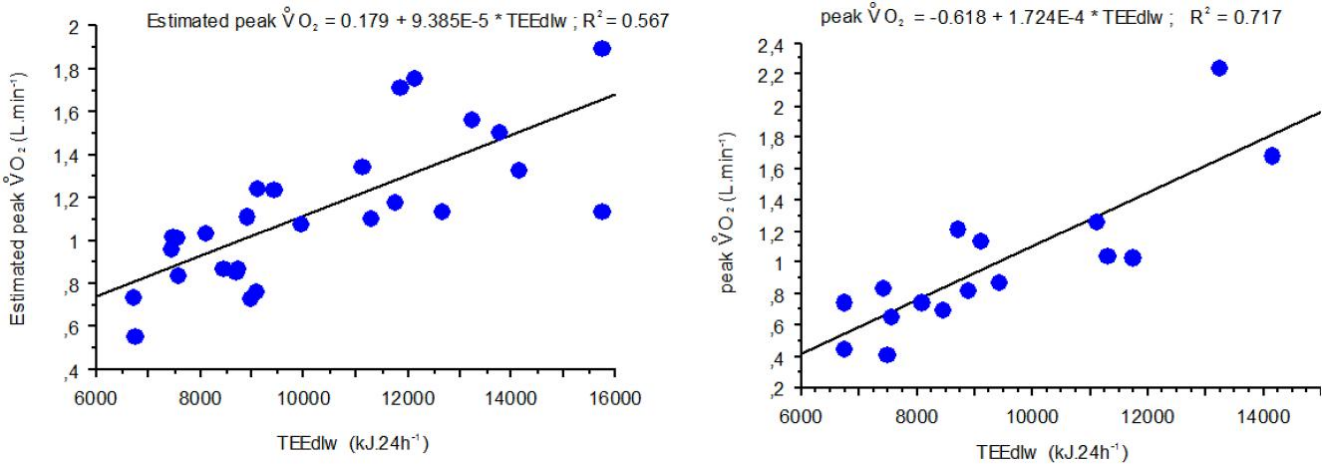
Relationship between measured and estimated peak V̇O_2_ and TEE_DLW_ in the whole population (*N* = 29).

Simple regression analyses performed separately indicated that both measured peak V̇O_2_ and estimated peak V̇O_2_ from the questionnaire were strongly correlated to TEE_DLW_ as shown in *Figure 3*.

### RMR measurements and estimation

No significant differences were found in RMR between RMR_calo_ and RMR_quest_ (5765.5 ± 1909.5 kJ.24h^−1^ vs. 6170.7 ± 1804.6 kJ.24h^−1^  *P* & NS) but RMR_BIA_ (6713.5 ± 1186.1 kJ.24h^−1^) as well as RMR_HB_ (7261.2 ± 975.9 kJ.24h^−1^) overestimated RMR_calo_ (+16.4%, *P* & 0.0028 and +25.9%, *P* & 0.0013). Significant differences in RMR appeared only between women and men for RMR_BIA_ (*P* & 0.0002) and RMR_HB_ (*P* & 0.0008) but were independent of NYHA class or ICM/DCM.

### Body composition, relationship to physical activity patterns

As presented in *Table 4*, significant differences were noticed between fat mass, fat-free mass or total body water when estimated from DLW and impedance. Body composition was very different between women and men but for fat mass expressed in kg (*P* = NS).

**Table 4:** Body composition variables estimated from BIA and DLW dilution techniques in the whole population (*N* = 29)

		TBW (L)	TBW (%)	FM (kg)	FM (%)	FFM (kg)	FFM (%)
DLW	Women (*n* = 12)	29.4 ± 3.8^a,b^	42.8 ± 3.8^a,b^	29.3 ± 8.7^b^	41.5 ± 5.2^a,b^	40.2 ± 5.2^a,b^	58.7 ± 5.5^a,b^
	Men (*n* = 17)	40.8 ± 6.5^a^	50.1 ± 3.9^a^	26.1 ± 7.7^c^	31.4 ± 5.4^a^	55.7 ± 8.9^a^	68.2 ± 5.4^a,c^
	Overall (*n* = 29)	32.9 ± 7.1*	45.1 ± 5.1*	28.3 ± 8.3*	38.3 ± 6.9*	45.1 ± 9.7*	61.6 ± 6.9*
BIA	Women (*n* = 12)	32.6 ± 2.9^a,b^	49.3 ± 6.6^a,b^	22.1 ± 7.2^b^	32.1 ± 5.3^a,b^	45.4 ± 5.3^a,b^	68.2 ± 5.6^a,b^
	Men (*n* = 17)	46.6 ± 3.5^a^	52.9 ± 2.5^a^	21.6 ± 2.7^c^	24.7 ± 3.3^a^	66.6 ± 5.5^a^	75.3 ± 3.3^a,c^
	Overall (*n* = 29)	36.9 ± 7.3*	50.6 ± 5.6*	21.9 ± 6.1*	29.8 ± 5.8*	51.9 ± 11.4*	70.4 ± 5.9*
							

BIA = body impedance analysis; DLW = doubly labelled water dilution technique; FM = for fat mass; FFM = fat-free mass; TBW = total body water.

*P < 0.05 between DLW and BIA analysis.

^a^ *P* < 0.05 between women and men.

^b^ *P* < 0.05 between DLW and BIA analyses in women.

^c^ *P* < 0.05 between DLW and BIA analyses in men.

Considering DLW dilution analysis as the referent method, single regression analyses showed that TEE_DLW_ as well as TEE_quest_ were correlated to total body water (*R*^2^ = 0.82, *P* < 0.0001; *R*^2^ = 0.62, *P* = 0.0004 respectively) and fat-free mass (*R*^2^ = 0.82, *P* < 0.0001; *R*^2^ = 0.61, *P* = 0.0004 respectively). Multiple regression analyses revealed that daily TEE and not a specific dimension of TEE was related to fat-free mass.

### Quality of life

The Duke quality of life questionnaire was assessed twice and no significant differences were noticed between the two assessments (*P* = NS). Mean quality of life scores reached 19 on the first assessment and were independent from sex, NYHA class, ICM/DCM and TEE, as well as from TEE dimensions.

## Discussion

The main results of this study indicate that the DAQIHF questionnaire allows a valid estimation of free-living TEE in patients with CHF when compared to the gold standard method, doubly labelled water. This is also true for the motion sensor in a subgroup of 12 women. Regarding the estimation of activity energy expenditure or in higher levels of activity (above 3 METs), it also appears that the questionnaire provides a valid estimation of energy spent when compared to data from validated motion sensors. Providing a valid estimation of TEE along with a complete picture of the patient’s habitual daily activities is of great interest in the promotion of an active preventive lifestyle through a precise follow-up of patients.

If the questionnaire allows an adequate evaluation of TEE, it also showed a high test-retest reliability for both TEE and TEE dimensions, confirming previous results.^18^ A difference with our previous study is that within the reproducibility study, even though valid, high-intensity activities reported by the patients appeared less reliable. As the time between the two assessments was shorter in this study, this modest reduction in reliability is probably due to the remembering of the patient and the willingness to report a more socially sought behaviour.

Peak *V’*O_2_ was correlated to TEE_DLW_ as well as TEE_quest_ in this study, which also confirms previous results.^13,18^ This further establishes the concurrent validity of the questionnaire, considering that the relationship between both factors is partially independent regarding the phenotype of the patient. Nonetheless, peak V̇O_2_ is a major integrative still considered as the best measure of physical fitness^6,20^ and widely used in clinical practice. An indirect valid estimation of peak V̇O_2_, as calculated from the questionnaire, since measured and estimated peak V̇O_2_ were similar, provides valuable information about clinical outcome, pathophysiological assessment and general health status of patients with CHF, even though its sole use is not sufficient in order to represent ‘real life’ condition.

TEE_quest_ using the factorial method that has proved to be valid in specific populations,^10,11,17–19,31–35^ was in agreement with previous studies measuring TEE_DLW_ in similar free-living patients with CHF.^13–15,18,19^ The corrections in the energy cost values made specifically for age, sex, autonomy, and severity of the disease, along with a minimized error in time reporting were thought in order to allow the best estimation of TEE for this tool. These correction coefficients are of particular importance in functionally limited population, as a calculation of TEE_quest_ without these coefficients led to a great overestimation of TEE, suggesting that this issue needs to be taken into account when evaluating TEE from a questionnaire.

However, it appears that along with the fair estimation of TEE against DLW, discrepancies at an individual level are important, ranging from −21.0% to +40.2% in TEE_quest_ for the whole population and from −21.4% to +32.4% in TEE_acti_ in women. These observations are in line with previous studies in other specific pathologic populations^10,32,33^ or in the elderly.^31,34,35^ This is clearly a limit of both methods. The previous results suggest that these discrepancies seem to be mainly due to a reduced reliability of energy expenditure estimation on low-intensity activities, both with questionnaires or motion sensors. The results of this study seem to confirm that both methods, questionnaire and motion sensor, present a good agreement in estimating energy expenditure for activities above three METs and for resting energy expenditure (compared to RMR_calo_ as the reference method), but show a significant difference in TEE, thus from energy quantification of activities between rest and three MET. Further studies exploring the energy cost of low-intensity activities in symptom-limited patients with chronic disease should be performed to improve the accuracy of both methods in estimating energy expenditure. Additionally, it should be acknowledged that motion sensor analysis was limited to female participants due to technical issues with sensor compliance and data quality in male participants, which represents a limitation in the generalizability of these findings across sex.

Several limitations of this study deserve mention. First, no formal *a priori* sample size calculation was performed. The sample size (*n* = 29) was based on previous validation studies using the DLW method in CHF populations,^13–15,19^ where comparable sample sizes were successfully employed. For the motion sensor subgroup (*n* = 12 women), while smaller, this represents all female participants with valid and reliable sensor data. Nevertheless, the relatively modest sample size, particularly for subgroup analyses, may limit the statistical power to detect smaller differences between methods and warrants cautious interpretation of findings. Second, as noted above, the motion sensor validation was restricted to women due to data quality issues in men, precluding sex-specific comparisons of this technology. Future studies with larger sample sizes and improved motion sensor protocols across both sexes would strengthen these findings.

Daily TEE decreased with NYHA class. This result confirms previous studies and is in line with a previous study from our team reporting a decrease in TEE with NYHA class, associated with a decrease in autonomic nervous system activity.^35^ This trend is consistent with the decrease in peak V̇O_2_ and with the relative sedentarity of this population reported in this study (9.4% of TEE spent in activities > 3 METs). As the severity of the condition definitely plays a major role in daily living activities, regular physical effort is recommended in this population, as the beneficial effects of an increase, even modest, in daily PA in secondary prevention appear essential.^9^ TEE was higher in men and the pattern of activities was different between women and men, with women being globally more active but spending more time and energy in low-intensity activities as compared to men. This is consistent with the differences in peak V̇O_2_ and body composition between men and women (*Table 4*). As formal intensive exercise rehabilitation programmes reduce rehospitalization rate and increase survival,^2–6,9^ moderate-to-intensive activities should be integrated into daily living activities in order to increase total energy expenditure. Providing the patient a complete quantified picture of his daily activities seems, therefore, appropriate. Regarding the results of this study, both the questionnaire and the motion sensor are appropriate to address this issue, but improvements in the individual quantification of TEE and PAEE should be performed.

### Conclusion

This study showed that, on average the DAQIHF showed a fair validity for estimating TEE across all activity domains in a population of patients with CHF. It also highlighted individual variations in the estimation of TEE, both with questionnaire and motion sensor, errors that should not be ignored in further analyses using these tools. Regarding its practical value, easiness of use, low cost, validity and ability to explore all domains of activity at various intensities, the DAQIHF seems a potentially useful tool for investigating PA and health-related issues in patients with CHF, particularly in large studies and/or clinical settings.

## Data Availability

The data underlying this article will be shared on reasonable request to the corresponding author.
